# ECMWF global coupled atmosphere, ocean and sea-ice dataset for the Year of Polar Prediction 2017–2020

**DOI:** 10.1038/s41597-020-00765-y

**Published:** 2020-12-02

**Authors:** Peter Bauer, Irina Sandu, Linus Magnusson, Richard Mladek, Manuel Fuentes

**Affiliations:** European Centre for Medium-Range Weather Forecasts, Reading, United Kingdom

**Keywords:** Physical oceanography, Atmospheric science

## Abstract

The Year Of Polar Prediction (YOPP) dataset of the European Centre for Medium-Range Weather Forecasts (ECMWF) contains initial condition and forecast model output from the operational global, coupled numerical weather prediction system. The dataset has been created to support model forecast evaluation, predictability studies and model error analyses over polar areas, which are strongly affected by climate change with yet unknown feedbacks on global circulation. The dataset complements YOPP observation and modeling research activities that represent a key deliverable of the World Meteorological Organization’s Polar Prediction Program. The dataset covers the period from mid-2017 until the end of the MOSAiC field campaign, expected for autumn 2020. Initial conditions and forecasts up to day-15 are included for the atmosphere and land surface for the entire period, and for ocean and sea-ice model components after June 2019. In addition, tendencies from model dynamics and individual physical processes are included for the first two forecast days. These are essential for characterizing the contribution of individual processes to model state evolution and, hence, for diagnosing sources of model error.

## Abstract (summary)

**Table:** 

Measurement(s)	temperature of air • air moisture • temperature of sea surface • water-based rainfall • ozone • pressure • atmospheric wind speed
Technology Type(s)	satellite imaging of a planet • weather station • computational modeling technique
Factor Type(s)	spectral space • year of data collection • hourly data collection
Sample Characteristic - Environment	atmosphere • ocean • sea • ice
Sample Characteristic - Location	global

Machine-accessible metadata file describing the reported data: 10.6084/m9.figshare.13013528

## Background & Summary

Given the amplified sensitivity of polar regions to climate change^1^ and its yet unknown effects on regional and even global weather patterns and their impacts on society and economies, there is a substantial need to make a concerted investment in polar research at international level. The World Meteorological Organization’s (WMO) Polar Prediction Project (PPP) under its World Weather Research Programme (WWRP) coordinates a major international polar research effort through its main flagship initiative, the Year of Polar Prediction (YOPP)^2,3^. The core phase of YOPP covered the period from July 2017 to July 2019. YOPP created the synopsis of dedicated polar field campaigns, enhanced operational observation programs and numerical experimentation to enhance our understanding of the Earth-system processes at high latitudes and to improve the representation of such processes in numerical models. Both the synergy between observation programmes and modeling and the focus on translating original research to operational benefit represent a significant step beyond the achievements of the International Polar Year efforts^4^ in 2007–2008.

The ongoing consolidation phase of YOPP (July 2019 to July 2023) aims to take advantage of the wealth of information gathered in the YOPP core phase to significantly advance the predictive skill of operational weather prediction systems and climate projections. The YOPP consolidation phase also overlaps with the MOSAiC field campaign^5^ supported by 17 nations, for which the icebreaker Polarstern drifts through the Arctic ice shield during winter 2019–2020 to collect a unique dataset on the physical and chemical properties of the coupled ocean-sea ice-atmosphere system under the most extreme conditions.

The YOPP dataset of the European Centre for Medium-Range Weather Forecasts (ECMWF) has been designed to (i) make freely available atmosphere, ocean and sea-ice output from the world’s leading global medium-range numerical weather prediction system and to (ii) add non-operational output that permits a deeper analysis of individual model process contributions to the evolution of state for identifying and characterising key sources of model error. The latter is crucial for achieving the overall goal of YOPP, namely the combined use of observations and simulations to advance model development and predictive skill from weather to climate scales. The dataset covers the period of the YOPP Core Phase and MOSAiC, i.e. July 2017 to October 2020, allowing to study several seasons in consecutive years.

In the past, a similar modelling effort had been made for the period May 2008 - April 2010 in support of the Year of Tropical Convection (YOTC)^6^. Since then, the YOTC dataset has supported a large number of scientific studies on tropical convection and its interactions with the large scale circulation, which are essential for enhancing predictive skill at sub-seasonal to seasonal scale. Following more than 10 years of scientific progress, the YOPP dataset makes available the output from a much improved atmosphere-land model that is coupled with an ocean and sea-ice model, is run at higher resolution and provides more comprehensive model output with higher frequency for detailed process studies. As the output is global the YOPP dataset will equally support research focusing on other regions and processes.

**Fig. 1:**
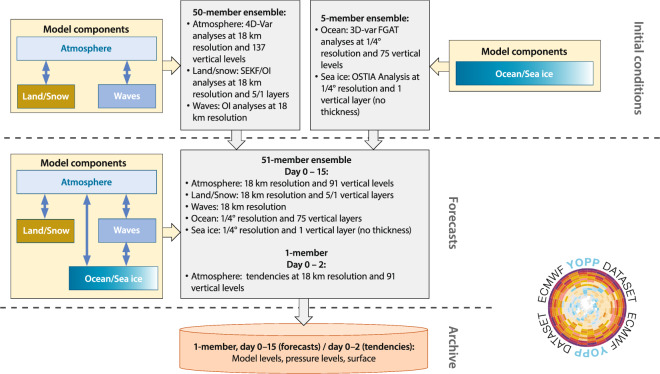
Schematic of the YOPP dataset production framework: The initial conditions and ensemble forecasts are generated by ECMWF’s operational forecasting system based on ensembles of data assimilation and models. Atmosphere, land and ocean waves are separate from the ocean and sea-ice circulation in the data assimilation system, while the forecasts are fully coupled. Only the unperturbed control experiment output is ingested in the YOPP dataset from day 0 to day 15, and complemented by physical process tendency output from day 0 to day 2.

The YOPP dataset has been established following the schematic shown in Fig. 1. The data is generated by the so-called ‘control’ forecast of the 51-member operational ECMWF ensemble and is the unperturbed, deterministic forecast of the ensemble. It contains 3-hourly output until day 7 followed by 6-hourly output until day 15, is issued twice per day, uses an 18-km resolution in the atmosphere/land-surface and 1/4 degree resolution in the ocean and sea-ice covered areas. A research experiment using the same set-up is run in parallel to the operational system and outputs additional fields for the first 48 hours containing tendencies of the atmospheric physical and dynamical processes with a 3-hour frequency. As the sum of these tendencies explains the change of state between consecutive model output times, the tendencies allow users to break down the overall change of state into the contribution from each process. The processes are shown in Table 1. Further details of dataset generation and contents are provided in the following section. Ocean and sea-ice model tendencies are not available.

**Table 1:** Main components and literature references for the atmosphere/land model IFS, the wave model WAM and ocean and sea-ice models NEMO and LIM.

Model component	References
Dynamical core	^18^^,34^
Shallow, mid-level and deep convection	^19–21^
Cloud microphysics	^22,23^
Planetary boundary layer and turbulence	^24,25^
Orographic gravity wave drag	^26,27^
Non-orographic gravity wave drag	^28^
Land surface	^29^^,35^
Radiation	^30^
Ocean waves	^31^
Ocean dynamics, physics, and sea ice	^32,33^

The code documentation can be found at https://www.ecmwf.int/en/publications/ifs-documentation.

## Methods

### Model output

The YOPP dataset combines operational forecast output with data from a research experiment using the same initial conditions and forecast set-up for storing model tendencies. The components of the operational forecasting system are listed in Table 1. The system consists of the Integrated Forecasting System (IFS) for the atmosphere and land-surface, the wave model (WAM), the Nucleus for European Modelling of the Ocean (NEMO, version 3.4) and the Louvain-la-Neuve sea-ice model (LIM, version 2).

The IFS is a spectral-transform model with prognostic variables being represented on both a cubic octahedral (reduced) Gaussian grid and in spectral space. The spectral representation facilitates gradient and derivative computations. Advection of constituents such as water vapour, condensed water, aerosols and trace gases is solved in grid-point space, and unresolved processes like convection, cloud microphysics, turbulence, radiation, land surface processes and their interaction are represented by parametrisations for each grid point and vertical column. The atmosphere model is then coupled to a wave model and models for ocean dynamics, physics and sea-ice processes allowing for two-way interactions.

The operational ECMWF coupled model evolves along update cycles, through which improvements to the representation of physical processes, the generation of the initial conditions and the use of observations in data assimilation are implemented. In the past, these improvements have led to a steady enhancement of medium-range predictive skill of about one forecast day per decade^7^. During the validity period of the YOPP dataset, four updates of the forecasting system have been introduced, namely model cycle 43r3 on 11 July 2017, 45r1 on 5 June 2018, 46r1 on 11 June 2019, and 47r1 on 30 June 2020 (https://www.ecmwf.int/en/forecasts/documentation-and-support/changes-ecmwf-model). This means that the YOPP dataset includes system changes, which can be delineated from the natural variability when compared, for example, to reanalysis datasets^8^ for which the prediction system remains unaltered throughout the application period.

The atmospheric component of the YOPP dataset is based on the ensemble (ENS) control forecast and output. Fields are gridded on the native octahedral model grid^9^ using the Meteorological Interpolation and Regridding (MIR) software package^10^.

The dataset is located in ECMWF’s MARS archive and has its own identifiers, namely class = yp, dataset = yopp using the ensemble control forecasts identifiers stream = enfo and type = cf. The full list of parameters, identifiers and units is shown in Table 2. The model tendencies use the identifiers stream = oper and type = fc. However, this information is not needed for data access through the interactive website. The data is stored in the GRIB-1 format^11^.

**Table 2:** YOPP dataset content description.

Type and levels	Time steps	Data volume per month in GBytes
*Forecasts atmosphere: 1 May 2017–31 October 2020* Model levels: 1 (model top) – 91 (model bottom)	3-hourly: 0–144 h, 6-hourly: 150–360 h	∼6,500
Pressure levels: 1, 2, 3, 5, 7, 10, 20, 30, 50, 70, 100, 150, 200, 250, 300, 400, 500, 600, 700, 850, 900, 925, 950, 1000 hPa	3-hourly: 0–144 h, 6-hourly: 150–360 h	∼1,700
Surface levels: 0–7 cm, 7–21 cm, 21–72 cm, 72cm-1.82 m	3-hourly: 0–144 h, 6-hourly: 150–360 h	∼1,800
*Forecasts tendencies atmosphere: (1 May 2017–31 October 2020)* Model levels: 1 (model top) – 91 (model bottom)	3-hourly: 0–48 h	∼3,300
Pressure levels: 1, 2, 3, 5, 7, 10, 20, 30, 50, 70, 100, 150, 200, 250, 300, 400, 500, 600, 700, 850, 900, 925, 950, 1000 hPa	3-hourly: 0–48 h	∼90
Surface levels	3-hourly: 0–48 h	∼156
*Forecasts ocean and sea ice: 12 June 2019–31 October 2020*	3-hourly: 0–144 h, 6-hourly: 144–360 h	∼0.5

The full parameter list and surface layer descriptions are shown at the bottom of this paper.

The NEMO and LIM model output is only available since 11 June 2019 as the capability of storing this output in the GRIB-1 format was only implemented with ECMWF’s model cycle 46r1. The YOPP dataset contains ocean and sea-ice data on a 1-degree latitude-longitude grid. Records of ocean and sea-ice data prior to 12 June 2019 need to be separately requested from ECMWF and can be made available in the NetCDF format. However, as the post-processing involved a different interpolation mechanism before cycle 46r1 these records have not been included here.

The relationship between model and pressure level data is explained through a simple relationship that uses surface pressure, *p*_*sfc*_, which is a prognostic variable of the hydrostatic IFS, such that *p*_*i*_ = *p*_*sfc*_*B*_*i*_ + *A*_*i*_ in units Pa. The coefficients *A*_*i*_ and *B*_*i*_ are static and defined for each set of model levels, here 91 levels (https://confluence.ecmwf.int/display/UDOC/L91+model+level+definitions). The process tendencies are accumulated values starting at the beginning of the forecasts, respectively. Thus the tendencies at forecast step 24 represent the accumulated tendencies over the first 24 hours of the model integration. This permits easy comparison of tendencies with overall state evolution of, for example, temperature between forecast step 0 and step 24 hours. To examine the tendencies, and model state evolution, between two consecutive output steps, i.e. 24 and 27, one needs to subtract the tendencies at step 24 from those at step 27. Note that the difference between states from consecutive model time steps is not exactly equal to the sum of the tendencies due to second order semi-Lagrangian averaging of tendencies along the trajectory^12^; however, given the output time steps are coarser this difference is negligible.

### Full parameter lists

The following list shows the complete set of parameters referred to in Table 2:

- Model and pressure level parameters, forecasts: divergence, fraction of cloud cover, logarithm of surface pressure, specific cloud ice water content, specific cloud liquid water content, specific humidity, specific rain water content, specific snow water content, temperature, vertical velocity, vorticity (relative), ozone mass mixing ratio, potential vorticity, relative humidity, stream function, u-component of wind, v-component of wind, velocity potential.
- Model level parameters, forecast tendencies: divergence, fraction of cloud cover, geopotential, ice precipitation flux from cloud scheme (stratiform), ice precipitation flux from convection, liquid precipitation flux from cloud scheme (stratiform), liquid precipitation flux from convection, logarithm of surface pressure, specific cloud ice water content, specific cloud liquid water content, specific humidity, specific rain water content, specific snow water content, temperature tendency from cloud scheme, temperature tendency from convection (deep + shallow), temperature tendency from dynamics, temperature tendency from radiation, temperature tendency from subgrid orography, temperature tendency from turbulent diffusion + subgrid orography, temperature, u-wind tendency from convection (deep + shallow), u-wind tendency from dynamics, u-wind tendency from subgrid orography, u-wind tendency from turbulent diffusion + subgrid orography, v-wind tendency from convection (deep + shallow), v-tendency from dynamics, v-wind tendency from subgrid orography, v-wind tendency from turbulent diffusion + subgrid orography, vertical velocity, relative vorticity, specific humifity tendency from cloud scheme, specific humidity tendency from convection (deep + shallow), specific humidity tendency from dynamics, specific humidity tendency from turbulent diffusion, ice water content tendency from cloud scheme, liquid water tendency from cloud scheme.

- Pressure level parameters, forecast tendencies: divergence, geopotential, relative humidity, specific humidity, temperature, u-wind component, v-wind component, vertical velocity, relative vorticity.
- Surface level parameters, forecasts: 2 metre dewpoint temperature, 2 metre temperature, 10 metre u-wind component, 10 metre v-wind component, 10 metre wind gust in the last 3 hours, 10 metre wind gust in the last 6 hours, 10 metre wind gust since previous post-processing, 100 metre u-wind component, 100 metre v-wind component, accumulated carbon dioxide ecosystem respiration, accumulated carbon dioxide gross primary production, accumulated carbon dioxide net ecosystem exchange, accumulated freezing rain, albedo boundary layer dissipation, boundary layer height ceiling, Charnock, clear-sky direct solar radiation at surface, cloud base height, convective available potential energy, convective available potential energy, shear, convective inhibition, convective precipitation, convective rain rate, convective snowfall rate water equivalent, direct solar radiation, downward UV radiation at the surface, eastward gravity wave surface stress, eastward sea water velocity, eastward turbulent surface stress, evaporation, flux of carbon dioxide, ecosystem respiration flux of carbon dioxide, gross primary production flux of carbon dioxide, net ecosystem exchange forecast, albedo forecast, logarithm of surface roughness for heat, forecast surface roughness, geopotential, gravity wave dissipation, height of convective cloud top, height of one-degree wet-bulb temperature, height of zero-degree wet-bulb temperature, high cloud cover, high vegetation cover, ice temperature layer 1, ice temperature layer 2, ice temperature layer 3, ice temperature layer 4, instantaneous 10 metre wind gust, instantaneous large-scale surface precipitation fraction, K index, lake bottom temperature, lake ice depth, lake ice temperature, lake mix-layer depth, lake mix-layer temperature, lake shape factor, lake total layer temperature, land-sea mask, large-scale rain rate, large-scale snowfall rate water equivalent, large-scale precipitation, low cloud cover, low vegetation cover, maximum temperature at 2 metres in the last 3 hours, maximum temperature at 2 metres in the last 6 hours, maximum temperature at 2 metres since previous post-processing, maximum total precipitation rate in the last 3 hours, maximum total precipitation rate in the last 6 hours, maximum total precipitation rate since previous post-processing, mean sea level pressure, medium cloud cover, minimum temperature at 2 metres in the last 3 hours, minimum temperature at 2 metres in the last 6 hours, minimum temperature at 2 metres since previous post-processing, minimum total precipitation rate in the last 3 hours, minimum total precipitation rate in the last 6 hours, minimum total precipitation rate since previous post-processing, northward gravity wave surface stress, northward sea water velocity, northward turbulent surface stress, potential evaporation, precipitation type, runoff sea ice area fraction, sea surface temperature, skin reservoir content, skin temperature, snow albedo, snow density, snow depth, snowfall, soil temperature level 1, soil temperature level 2, soil temperature level 3, soil temperature level 4, soil type, sub-surface runoff, sunshine duration, surface latent heat flux, surface net solar radiation, surface net solar radiation clear sky surface net thermal radiation, surface pressure, surface roughness, surface runoff, surface sensible heat flux, surface solar radiation downwards, surface thermal radiation downwards, temperature of snow layer, top net solar radiation, top net thermal radiation, total cloud cover, total column cloud ice water, total column cloud liquid water, total column ozone, total column rain water, total column snow water, total column super-cooled liquid water, total column water, total column water vapour, total precipitation, total sky direct solar radiation at surface, type of high vegetation, type of low vegetation, visibility, volumetric soil water layer 1, volumetric soil water layer 2, volumetric soil water layer 3, volumetric soil water layer 4, zero degree level.

Land surface and ice parameter layers are 1: 0–7 cm, 2: 7–21 cm, 3: 21–72 cm, 4: 72cm-1.82 m for volumetric soil water, soil temperature and ice temperature layer.

- Ocean and sea-ice model parameters, forecasts: average potential temperature in the upper 300 m, average salinity in the upper 300 m, depth of 20 C isotherm, eastward sea water velocity, northward sea water velocity, sea surface height, sea water practical salinity, sea-ice thickness.

## Data Records

The YOPP dataset can be accessed through 10.21957/mqze-0a53^13^, and its list of contents is shown in Table 2 and the Appendix. The data are available for the periods indicated in the table. The operational ENS forecasts, from which the YOPP forecasts are extracted, are issued at 0 and 12 UTC every day, respectively. Time step 0 represents the initial conditions produced by the analysis and is included in the dataset. The analysis can serve for forecast verification as shown in the Validation Section. These analyses are used to initialise both ENS and the so-called high-resolution, deterministic forecasts (HRES). The model tendencies are only archived for the forecasts starting at 0 UTC. As of April 2020, the total archived volume has reached 472 TBytes with over 285 million fields.

This paper’s figures are available from the figshare repository^14^.

## Technical Validation

It is worth noting the differences between the YOPP dataset described here and the THORPEX Interactive Grand Global Ensemble (TIGGE) dataset that includes ensemble output provided by several global modeling centres for performance intercomparison and case study research^15^.

The TIGGE dataset is based on the IFS ENS as well but has much more limited number of parameters (25 surface and 5 atmospheric parameters on 9 pressure levels), and data is only stored in 6-hourly time steps up to day 15 (https://apps.ecmwf.int/mars-catalogue/?class=tiexpver=prodmodel=globtype=cfyear=2020month=maylevtype=sfcdate=2020-05-01time=00:00:00).

### Example: Forecast errors

**Fig. 2:**
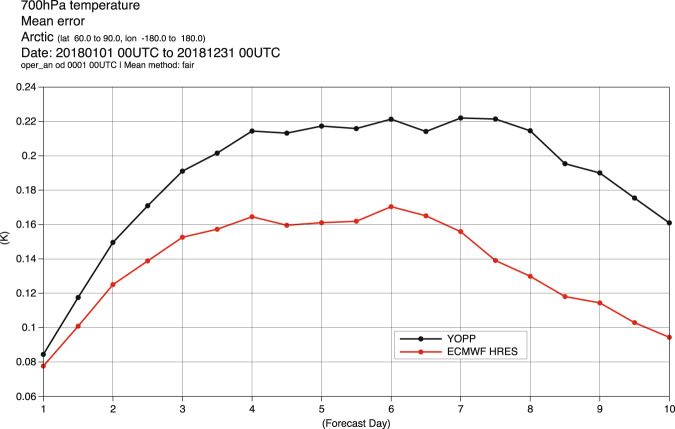
Mean error as a function of lead time for 700hPa temperature forecasts for 2018 averaged over the Arctic. The ECMWF HRES forecast (red) and the ENS control forecast used in the YOPP dataset (black). Verification is performed against the operational ECMWF analysis (included as time step 0).

Figure 2 shows the mean error for the 700 hPa temperature averaged of the Arctic (60 N–90 N) for the full year of 2018. The plot includes the results for the ECMWF HRES (red) and the ENS control forecast that is archived in the YOPP data set (black). Both forecasts show a warm bias at this level that amplifies throughout the first forecast days. The YOPP forecast has a larger bias than HRES, but the similarities in the error indicates that lessons learnt from this dataset on sources of such errors can also be translated to the ECMWF HRES forecasts. We also see that the error has a significant amplitude already at forecast day 1, which means that by studying short-range forecast errors we can learn about medium-range errors.

### Example: Tendency analysis

**Fig. 3:**
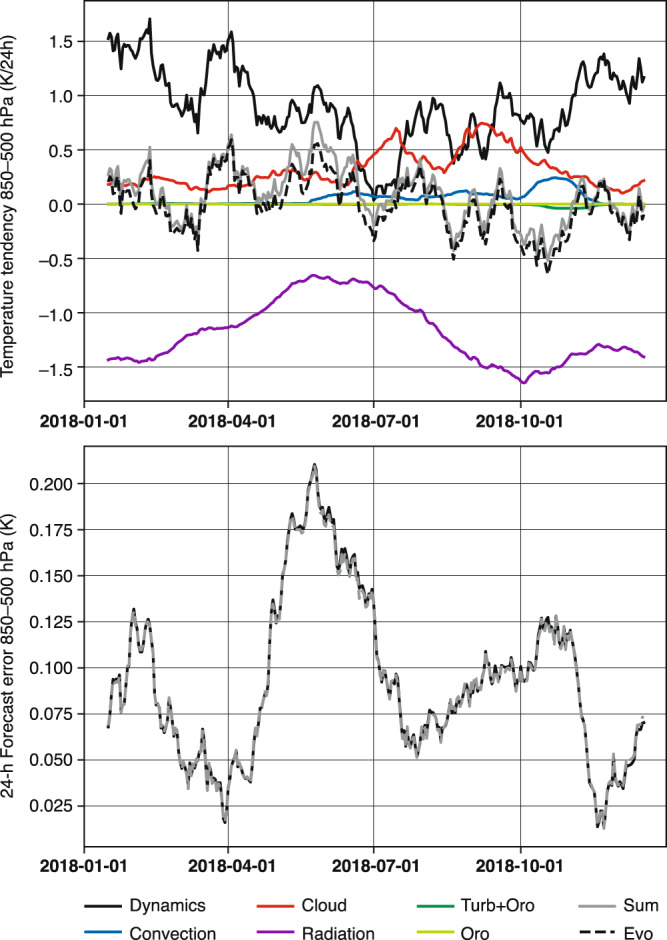
Time series of model tendencies for temperature averaged over the first 24 forecast hours (top) and 24-hour temperature forecast errors (bottom), averaged between 850 hPa and 500 hPa. The top panel also includes the evolution of the temperature over 24 hours. The bottom panel includes the forecast error calculated from the 24-hour forecast output (black) and from the sum of the tendencies minus the evolution (grey dashed). A 30-day running mean is applied to the time-series. All data is averaged inside a box between 75N-85N latitude and 110E-170E longitude.

**Fig. 4:**
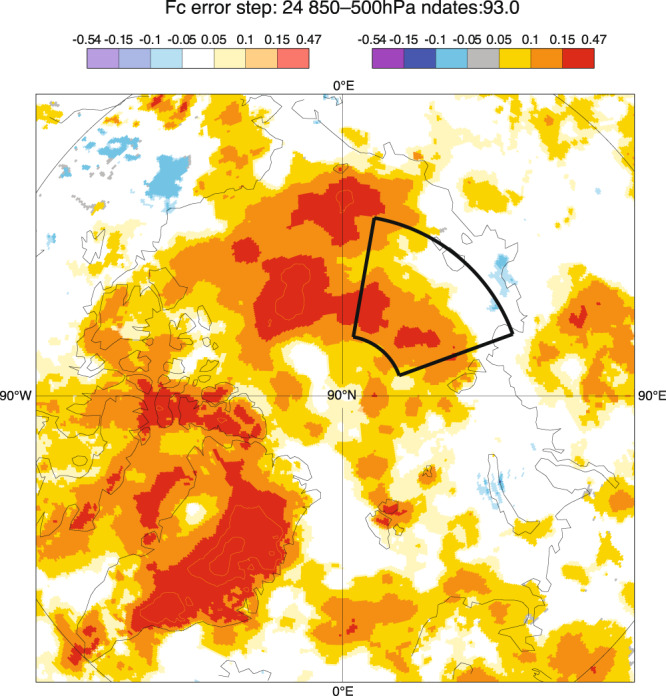
24-hour temperature forecast errors, averaged between 850 hPa and 500 hPa for the period 1 July to 1 October 2018. Darker colours (top right colour bar) indicate statistical significance at the 95% level. Lighter colours (top left colour bar) indicate data where statistical significance is insufficient; this only applies to white areas.

Figure 3 shows the time series of model tendencies for temperature (top) averaged over 24 hours and the 24-hour forecast errors (bottom), with a 30-day running mean applied. The data is averaged inside the box over the Eastern Arctic (75N-85N, 110E-170E), outlined in Fig. 4, and between model levels 77 to 81, which roughly corresponds to 500 and 850 hPa or 1 km and 5 km altitude. In the Arctic, this represents most of the free troposphere.

The top panel also includes a curve for the temperature evolution between two consecutive analyses (=forecast step 0), separated by 24 hours (black-dashed). If the forecast would be perfect, the sum of the tendencies (which govern the forecast evolution, grey line) should be the same as the temperature evolution between the two analyses (under the assumption that the analyses are also perfect). The figure shows numerical noise effects, which can be locally large (in time and space) but are in general negligible on average over a region/longer period^16^. Neglecting this effect, the difference between the grey and dashed black lines represents the forecast error (see grey-dashed line in bottom panel).

For the tendencies, the dynamic tendencies (black) are positive if warmer air is advected into the atmosphere segment the averaging is performed for. Generally speaking, air is loosing energy by long-wave radiative cooling (purple). Latent heat release through condensation in clouds results in a positive temperature tendency (red), which also occurs in convection (blue). If there is a net evaporation of condensed water in the volume, the overall tendencies from these schemes are negative. Finally, mixing of air due to turbulence and orographic/non-orographic gravity waves can contribute to both net heating and cooling. However, these terms are small in this example, given that the free-troposphere is largely stably stratified and the selected region is far from mountainous regions.

In the Arctic, the two largest terms are the dynamic and radiative tendencies in the free troposphere because warm air is advected into the Arctic, and at the same time experiences radiative cooling. More generally, cloud process contributions are strongest in summer. During the autumn when the water is still open, i.e. not covered by sea ice, cold air outbreaks from the north can lead to the formation of convective clouds, which amplifies convection tendencies. The dataset will stimulate detailed analyses of the link between potential sources of model errors in individual processes and their impact on predictive skill at local, regional and global scale - which is relevant for both weather and climate research^2,17^.

The mean error in the selected atmospheric volume is positive, with the largest amplitude in late spring/early summer. The active processes during the time are the advection by the dynamics and cloud condensation/evaporation that have a net warming tendency and the radiation that has a net cooling tendency. One could speculate that the radiation effect by the clouds plays a role for the development of the error.

Figure 4 shows the spatial pattern of 24-hour forecast error for July, August and September, averaged between 850–500hPa heights. The error is calculated as the difference between 24-hour forecasts and the verifying analysis. By using the initial conditions for the forecast (the analysis) as ‘truth’, one needs to be aware of the caveats in terms of analysis errors and the correlations with short forecast errors.

For this period, the temperature errors are positive over the Arctic basin, which is consistent with the errors inside the domain shown in Fig. 3.

## Data Availability

The IFS forecast model and the Meteorological Archival and Retrieval System (MARS) software are not available for public use as the ECMWF Member States are the proprietary owners. However, the YOPP dataset and the MARS data extraction features are freely available through the YOPP dataset website’s API (https://apps.ecmwf.int/datasets/data/yopp/levtype=sfc/type=cf/) following a registration step.
